# Geosocial networking mobile applications use and HIV and other sexually transmitted infections among men who have sex with men in Southern China: A cross-sectional study

**DOI:** 10.3389/fpubh.2023.1063993

**Published:** 2023-02-08

**Authors:** Zhihui Guo, Anping Feng, Yiguo Zhou, Yanxiao Gao, Yinghui Sun, Yuanyi Chen, Xinyi Zhou, Huachun Zou

**Affiliations:** ^1^School of Public Health (Shenzhen), Sun Yat-sen University, Shenzhen, China; ^2^Department of Laboratorial Science and Technology & Vaccine Research Center, School of Public Health, Peking University, Beijing, China; ^3^Kirby Institute, University of New South Wales, Sydney, NSW, Australia

**Keywords:** MSM, GSN apps, HIV, STIs, high-risk sexual behaviors

## Abstract

**Introduction:**

Men who have sex with men (MSM) are increasingly using geosocial networking (GSN) mobile applications (apps) to socialize in the community. Our study aimed to compare sexual behaviors between app-using MSM (app users) and non-app-using MSM (non-app users), and evaluate the association between app use and sexually transmitted infections (STIs).

**Methods:**

Eligible MSM were recruited from January to August 2017 in three metropolitan cities: Guangzhou, Shenzhen and Wuxi. A self-completed tablet-based questionnaire was collected about socio-demographic characteristics, sexual behaviors and app use. Blood samples were collected to test for HIV and syphilis. Rectal swabs taken by nurses and urine samples taken by participants themselves were collected to test for gonorrhea and chlamydia. Anogenital warts were checked by a clinician. Chi square tests and logistic regression were used to compare the prevalence of STIs and the characteristics between app users and non-app users.

**Results:**

A total of 572 MSM were included in our analysis, 59.9, 25.7, and 23.4% MSM were recruited from Guangzhou, Shenzhen, and Wuxi, respectively. The majority of participants were 20–29 years old (61.7%). 89.0% of MSM had ever used at least one GSN app, and 63.8% MSM had anal intercourse (AI) partners found *via* apps. Among app users, 62.7% spent <30 min on apps per day on average in the past 6 months. Compared with non-app users, app users were more likely to have an education level of college and above [adjusted OR (AOR) 3.36, 95% confidence interval (CI) 1.65–7.03], have regular sex partners (2.40, 1.16–5.19), have two or more casual sex partners (2–5: 2.90, 1.21–6.90; ≥6: 13.91, 3.13–82.90), have condomless anal intercourse (CAI) with casual sex partners in the past 6 months (2.50, 1.28–5.04), do not know their last sex partners' HIV status (2.16, 1.13–4.21), have tested for HIV in the past year (2.09, 1.07–4.09) and be circumcised (4.07, 1.29–18.42). Prevalence of HIV (8.3 vs. 7.9%, *P* = 0.93), syphilis (6.9 vs. 11.1%, *P* = 0.34), gonorrhea (5.1 vs. 6.3%, *P* = 0.90), chlamydia (18.5 vs. 12.7%, *P* = 0.36), and anogenital warts (4.9 vs. 4.8%, *P* = 1.00) were similar between app users and non-app users.

**Conclusions:**

GSN app users were more likely to have high-risk sexual behaviors, but the prevalence of HIV and other STIs were similar to non-app users. Longitudinal studies comparing the incidence of HIV/STIs between long-term app users and non-app users may be necessary to clarify the impact of app use on HIV/STIs risk.

## 1. Introduction

Men who have sex with men (MSM) are at high-risk for human immunodeficiency virus (HIV) infection and other sexually transmitted infections (STIs) in China and worldwide (1). Statistics showed that MSM accounted for about a quarter (23.3%) of the national new HIV cases in China in 2020 (2). The prevalence of HIV among MSM has been rising rapidly, with the percentage increasing from 1.4% in 2005 to 8% in 2020 (3, 4). A recent meta-analysis reported the prevalence of syphilis, gonorrhea, chlamydia in MSM were 11.8, 1.9, and 6.3% in China (5, 6), which were much higher than 0.5, 0.2, and 3.0% among general population (6). MSM tend to engage in high-risk sexual behaviors, such as low rate of condom use in anal sex, multiple casual sex partners, group sex and drug use during sex (7), which increases the risk of HIV and other STIs transmission among MSM.

In the past decades, the development of the Internet and the popularity of global positioning system (GPS)-equipped smartphone have drastically changed the ways of seeking sexual partners among MSM (8–11). MSM have moved away from the traditional venues to the geosocial networking (GSN) mobile applications (apps) (12–14). GSN apps, such as Grindr, Blued, Jack'd, allow members to create individualized profiles, chat, share photos, and send their exact location (13–15). Users can identify nearby users, which simplifies the process of identifying potential sex partners (7). GSN apps are widely used by MSM to socialize in the community. Previous studies estimated that up to 80% of MSM used GSN apps to seek sex partners worldwide (7, 13), and 66.0% in China in 2016 (16). And the largest GSN app, Blued (17, 18), has around 54 million registered users worldwide (19).

With the proliferation of apps, increased use of apps may facilitate sex partners seeking. This aroused much concern over its association with high-risk sexual behaviors, which may further lead to the infection of HIV and other STIs. However, the results of previous studies are inconsistent. Some work indicated that app-using MSM (app users) were associated with multiple sex partners (9, 16, 20–22), condomless anal intercourse (CAI) (16, 22, 23), group sex (8, 21, 24), and drug use (9, 20, 21), which could increase their risk of HIV infection (16, 21, 25). Whereas, some other studies suggested that app users were more likely to use condoms and routinely test for HIV (9, 12, 21, 26, 27), and the prevalence of HIV was similar between app users and non-app users.

To our knowledge, the majority of prior studies which investigated app use among MSM in China were online self-administered surveys (12, 16, 23, 27), which did not ensure that participants correctly understood the questions. The reliability of data collected may be compromised. We collected information with the assistance of investigators, which ensured the quality of data. Meanwhile, prior studies did not investigate app users' sexual behaviors of regular and casual sex partners and HIV status of participants were self-reported (12, 23). And few previously published studies investigated GSN app use and its association with STIs other than HIV among MSM that were based on laboratory testing of STIs in China.

The purposes of this study were to compare sexual behaviors between app users and non-app users, and evaluate the association between app use and HIV and other STIs.

## 2. Materials and methods

### 2.1. Subjects and recruitment

MSM were recruited between January and August 2017 *via* MSM community organizations and the study was conducted at three sexual health clinics in three metropolitan cities in southern China: Guangzhou, Shenzhen and Wuxi. Subjects were recruited in three ways: (1) investigators put on recruitment posters in three sexual health clinics; (2) doctors encouraged participants to introduce the program to their peers; (3) local MSM community organizations recommended potentially eligible subjects to participate in this program. This study is part of the baseline survey of the T2T study, which is a randomized controlled trial that evaluates the impact of automated text message reminders on HIV or sexually transmitted disease (STD) testing behaviors among MSM in China. Details of study design of the T2T Study were published elsewhere (1).

The criteria for recruiting were (1) physiological male aged 18 years or older; (2) had anal intercourse with a male partner in the past 6 months; (3) willing to provide informed consent for the data collection and testing for HIV/STIs. The exclusion criteria of this study were (1) severe psychiatric illnesses; (2) unable to read or use the iPad questionnaire; and (3) unable to speak or read Mandarin.

Participants interested in our study were invited to see the study research assistant, who took them to a private room where an iPad questionnaire system is installed. The study research assistant explained what the study involves and ask participants to complete the iPad-based questionnaire on their own. Participants who had any further questions could ask the research assistant who were outside of the private room.

### 2.2. Questionnaire

All data were collected through a tablet-based questionnaire. The questionnaire collected information as follows: (1) socio-demographic information including year of birth, marital status, time of stay in local city, education, income and profession; (2) sexual behavior characteristics in the past 6 months, including age and sexual role of anal sex debut, casual sex partners (who have casual sexual relationships with participants for < 3 months), regular sex partners (who have regular sexual or romantic relationships with participants for at least 3 month), female sex partners, and CAI; (3) patterns of GSN apps user (who has download GSN apps and registered for an account), including login time, time spent on apps per day in the past 6 months, number of anal intercourse (AI) partners found through apps in the past 6 months and condom use with them. Participants were also asked about HIV testing and whether performed circumcision.

### 2.3. Measures

The dependent variable was whether the MSM were app users, measured by the item “Have you ever used GSN apps such as Blued and Jack'd?” (Yes, or No). App users were defined as MSM who have download GSN apps and registered for an account. The independent variable of regular sex partners was measured by the item “Do you have regular sex partners (who have regular sexual or romantic relationships with you for at least 3 month) at present?” (Yes, or No), while casual sex partners was measured by the item “How many casual sex partners (who have casual sexual relationships with you for <3 months, such as one night stand) did you have in the past 6 months?” (Answer with a number).

### 2.4. Specimen collection and STIs testing

For all eligible participants, 5 ml of blood was collected for HIV and syphilis tests, and a nurse collected a rectal swab to test for rectal gonorrhea and chlamydia. A urine sample was collected by participants themselves to test for urethral gonorrhea and chlamydia. HIV was preliminarily screened by ARCHITECT HIV Ag/Ab Combo assay (Abbott Laboratories, Abbott Park, Illinois, USA) and positive samples were followed by enzyme linked immunosorbent assay (ELISA; Bio-Rad Laboratories, California, USA) for further screening. For positive specimens for ELISA, HIV1/2 western blot assay (HIV Blot 2.2WB; Gene labs Diagnostics, Singapore) was used for final confirmation. Syphilis was tested by the toluidine red unheated serum test (TRUST; RSbio, Shanghai, China), with the positive samples further confirmed by treponema pallidum particle assay (TPPA; Fuji Re Bio, Tokyo, Japan). PCR (Roche Diagnostics, Shanghai, China) was used to test for rectal and urethral gonorrhea and chlamydia. Anogenital warts were diagnosed by a doctor with the acetic acid test.

### 2.5. Statistical analysis

Categorical variables were presented as frequency and percentage. Chi square tests were used to compare the prevalence of STIs between app users and non-app users. Univariate and multivariate logistic regression models were used to compare the characteristics between app users and non-app users. To identify the significant factors associated with app use, variables with *P* < 0.10 in the univariate logistic regression models and theoretically important covariates were included in multivariate regression models and selected using a stepwise method. Adjusted ORs (AORs) and 95% confidence intervals (CIs) were calculated. Two-sided *P*-values < 0.05 was considered statistically significant. All analyses were performed using R (V.3.6.1, Foundation for Statistical Computing, Vienna, Austria).

### 2.6. Ethical issues

The study has been approved by the ethical review committees of the University of New South Wales, Australia (HC16803), the Dermatology Hospital of Southern Medical University (GDDHLS-20160926) and Wuxi Center for Disease Control and Prevention (WXCDC2016009), China. Informed consent was obtained from each participant before the study. The original informed consent allows the secondary analyses without additional consent. Study participants received health education materials, condoms and lubricant on completion the questionnaires. Participants who were diagnosed as STIs were referred for treatment.

## 3. Results

### 3.1. Demographics and behaviors

A total of 603 MSM were recruited. Among these, 28 were excluded because they did not have anal sex in the past 6 months, three refused to provide biological samples. Thus 572 participants were included in the analysis. 59.9% (291/572), 25.7% (147/572), and 23.4% (134/572) MSM were recruited from Guangzhou, Shenzhen, and Wuxi, respectively. The median age of participants was 27, 27, and 22 years in Guangdong, Shenzhen, and Wuxi. The majority of participants were 20–29 years old (61.7%), were single/divorced/widowed (64.0%), and had an education level of college and above (67.0%). And 53.7% (307/572) of participants had regular sex partners, 65.2% (373/572) had two–five casual sex partners in the past 6 months, with 82.7% (254/307) had CAI with regular sex partners and 53.1% (274/516) had CAI with casual sex partners. Among all participants, 54.7% (313/572) did not know their last sex partner's HIV status, and 73.6% (421/572) had tested for HIV in the past year (Table 1).

**Table 1:** Univariate and multivariate logistic analyses of characteristics of GSN app users and non-app users (*N* = 572).

	**App-using MSM** **(*n* = 509)** ***n*** **(%)**	**Non-app-using MSM** **(*n* = 63)** ***n*** **(%)**	**Total** **(*N* = 572)** ***n*** **(%)**	**OR (95%CI)**	**AOR (95%CI)**
**Age, year**					
<20	37 (7.3)	17 (27.0)	54 (9.4)	1	1
20–29	321 (63.1)	32 (50.8)	353 (61.7)	4.61 (2.31–9.04)^***^	3.04 (1.22–7.55)^*^
≥30	151 (29.7)	14 (22.2)	165 (28.8)	4.96 (2.25–11.12)^***^	2.93 (1.09–8.10)^*^
**Marriage**					
Married/cohabitation with a woman	63 (12.4)	10 (15.9)	73 (12.8)	1	–
Cohabitation with a man	125 (24.6)	8 (12.7)	133 (23.3)	2.48 (0.93–6.79)	–
Single/divorced/windowed	321 (63.1)	45 (71.4)	366 (64.0)	1.13 (0.52–2.28)	–
**Time of residence in the recruited city**					
≤ 1 year	115 (22.6)	24 (38.1)	139 (24.3)	1	–
>1 year	394 (77.4)	39 (61.9)	433 (75.7)	2.11 (1.20–3.63)^**^	–
**Education**					
High school and below	149 (29.3)	40 (63.5)	189 (33.0)	1	1
College and above	360 (70.7)	23 (36.5)	383 (67.0)	4.20 (2.45–7.36)^***^	3.36 (1.65–7.03)^**^
**Salary (RMB)** ^a^					
< 2,000	56 (11.0)	8 (12.7)	64 (11.2)	1	–
2,000–4,999	157 (30.8)	20 (31.7)	177 (30.9)	1.12 (0.44–2.61)	–
5,000–9,999	211 (41.5)	27 (42.9)	238 (41.6)	1.12 (0.45–2.49)	–
≥10,000	85 (16.7)	8 (12.7)	93 (16.3)	1.52 (0.53–4.35)	–
**Student**					
No	459 (90.2)	58 (92.1)	517 (90.4)	1	–
Yes	50 (9.8)	5 (7.9)	55 (9.6)	1.26 (0.53–3.75)	–
**Sexual role at anal sex debut**					
Insertive	155 (30.5)	38 (60.3)	193 (33.7)	1	1
Receptive	251 (49.3)	18 (28.6)	269 (47.0)	3.42 (1.91–6.33)^***^	2.97 (1.45–6.35)^**^
Insertive and receptive	103 (20.2)	7 (11.1)	110 (19.2)	3.61 (1.64–9.10)^**^	4.44 (1.67–13.72)^**^
**Regular sex partners**					
No	228 (44.8)	37 (58.7)	265 (46.3)	1	1
Yes	281 (55.2)	26 (41.3)	307 (53.7)	1.75 (1.04, 3.01)^*^	2.40 (1.16–5.19)^*^
**Number of casual sex partners** ^b^					
≤ 1	114 (22.4)	22 (34.9)	136 (23.8)	1	1
2–5	335 (65.8)	38 (60.3)	373 (65.2)	1.70 (0.95–2.98)	2.90 (1.21–6.90)^*^
≥6	60 (11.8)	3 (4.8)	63 (11.0)	3.86 (1.27–16.77)^*^	13.91 (3.13–82.90)^**^
**Sex experience with women**					
No	357 (70.1)	27 (42.9)	384 (67.1)	1	–
Yes	152 (29.9)	36 (57.1)	188 (32.9)	0.32 (0.19–0.54)^***^	–
**CAI with regular sex partners** ^b^					
No	49 (17.4)	4 (15.4)	53 (17.3)	1	–
Yes	232 (82.6)	22 (84.6)	254 (82.7)	0.86 (0.24–2.37)	–
**CAI with casual sex partners** ^b^					
No	209 (45.2)	33 (61.1)	242 (46.9)	1	1
Yes	253 (54.8)	21 (38.9)	274 (53.1)	1.90 (1.08–3.43)^*^	2.50 (1.28–5.04)^**^
**HIV status of the last sex partner**					
Negative	204 (40.1)	35 (55.6)	239 (41.8)	1	1
Positive	18 (3.5)	2 (3.2)	20 (3.5)	1.54 (0.42, 9.98)	3.96 (0.50–88.97)
Unknow	287 (56.4)	26 (41.3)	313 (54.7)	1.89 (1.11, 3.27)^*^	2.16 (1.13–4.21)^*^
**Ever rush popper use**					
No	387 (76.0)	57 (90.5)	444 (77.6)	1	–
Yes	122 (24.0)	6 (9.5)	128 (22.4)	8.15 (6.31–10.71)^***^	–
**Circumcision**					
No	421 (82.7)	59 (93.7)	480 (83.9)	1	1
Yes	88 (17.3)	4 (6.3)	92 (16.1)	3.08 (1.23–10.35)^*^	4.07 (1.29–18.42)^*^
**HIV test in the past year**					
No	124 (24.4)	27 (42.9)	151 (26.4)	1	1
Yes	385 (75.6)	36 (57.1)	421 (73.6)	2.33 (1.35, 3.98)^**^	2.09 (1.07–4.09)^*^
**Any HIV/STIs**					
Negative	341 (67.0)	43 (68.3)	384 (67.1)	1	–
Positive	168 (33.0)	20 (31.7)	188 (32.9)	0.84 (0.61–1.89)	–

* *P* < 0.05.

** *P* < 0.01.

*** *P* < 0.001.

OR, odds ratio; AOR, adjusted odds ratio; AI, anal intercourse; CAI, condomless anal intercourse.

a US$1=6.39 Chinese yuan renminbi (RMB) in 2021.

b In the past 6 months.

### 3.2. App use

**Figure 1:**
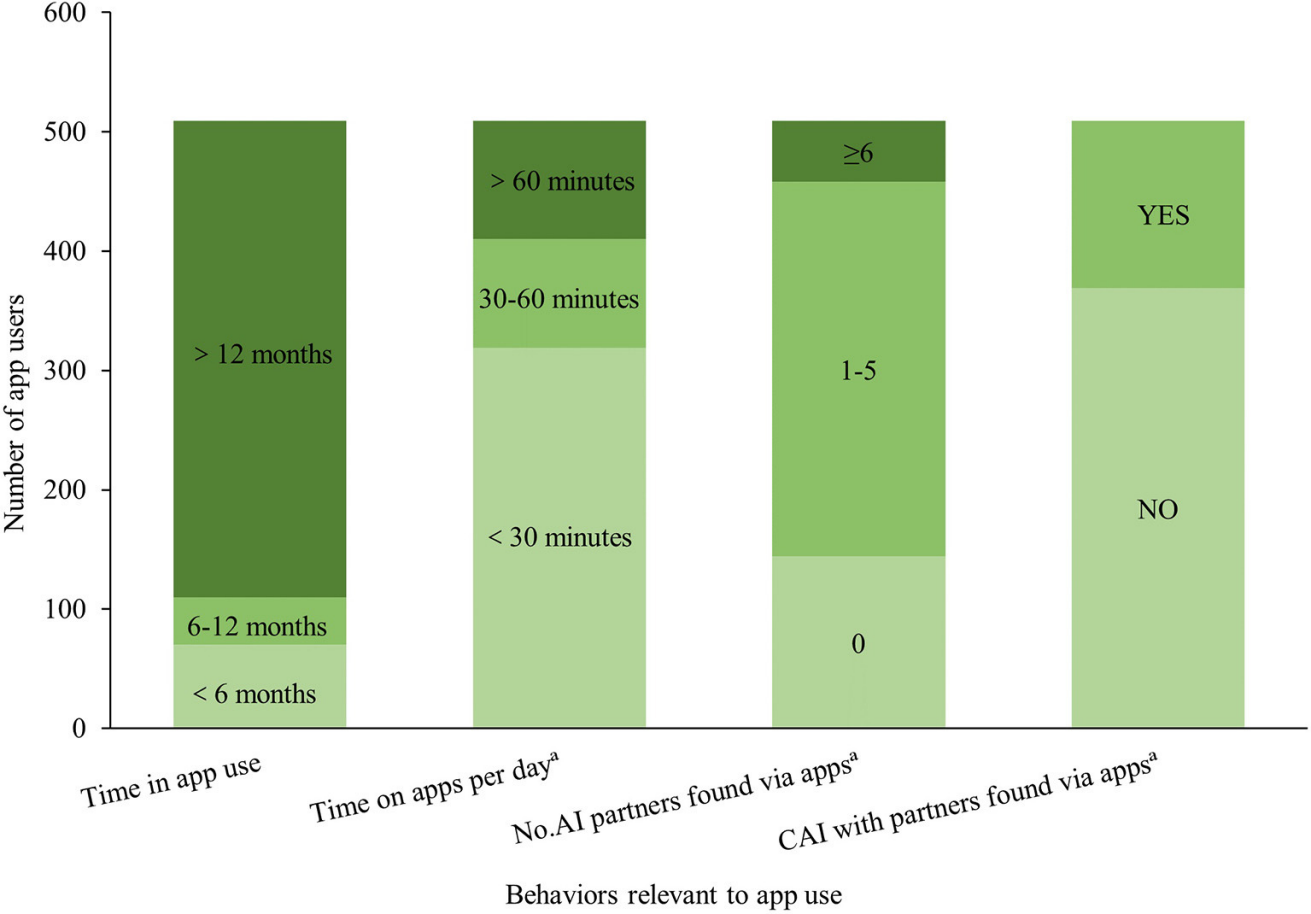
Self-reported GSN app use among MSM (*N* = 509). AI, anal intercourse; CAI, condomless anal intercourse. ^a^In the past 6 months.

89.0% (509/572) MSM had ever used at least one GSN app, the proportions were 93.8% (273/291), 94.6% (139/147), and 72.4% (91/134) in Guangdong, Shenzhen, Wuxi, respectively. And 63.8% (365/572) MSM had AI partners found *via* apps in the past 6 months. Among app users, 78.4% (399/509) had used apps for more than 12 months, 62.7% (319/509) spent <30 min on apps per day on average, 61.7% (314/509) had one–five AI partners found *via* apps, and 27.5% (140/509) had CAI with partners found *via* apps, in the past 6 months (Figure 1).

### 3.3. Factors associated with app use

Univariate and multivariate logistic analyses of characteristics of app use are presented in Table 1. In the multivariable analysis, app users had higher odds of having educational level of college and above (AOR 3.36, 95% CI 1.65–7.03), playing receptive (2.97, 1.45–6.35) or both receptive and insertive role (4.44, 1.67–13.72) at anal sex debut, having regular sex partners (2.40, 1.16–5.19), having 2–5 (2.90, 1.21–6.90), or ≥6 casual sex partners (13.91, 3.13–82.90), having CAI with casual sex partners in the past 6 months (2.50, 1.28–5.04), and not knowing last sex partners' HIV status (2.16, 1.13–4.21). In the meantime, circumcision (4.07, 1.29–18.42) and HIV testing rate in the past year (2.09, 1.07–4.09) were much higher among app users.

### 3.4. Prevalence of STIs

Details of the prevalence of HIV and other STIs are shown in Table 2. No statistically significant difference was found in the overall prevalence of HIV/STIs (33.0 vs. 31.7%, *P* = 0.84) comparing app users and non-app users. The infection rates of HIV (8.3 vs. 7.9%, *P* = 0.93), syphilis (6.9 vs. 11.1%, *P* = 0.34), gonorrhea (5.1 vs. 6.3%, *P* =0.90), chlamydia (18.5 vs. 12.7%, *P* = 0.36), and anogenital warts (4.9 vs. 4.8%, *P* = 1.00) were similar between app users and non-app users.

**Table 2:** STIs of GSN app users and non-app users (*N* = 572).

**Total (*****N*** = **572)** ***n*** **(%)**		**App-using MSM** **(*n* = 509)** ***n*** **(%)**	**Non-app-using MSM** **(*n* = 63)** ***n*** **(%)**	**χ^2^**	***P*** **-value**
**HIV**				0.007	0.93
Negative	525 (91.8)	467 (91.7)	58 (92.1)		
Positive	47 (8.2)	42 (8.3)	5 (7.9)		
**Syphilis**				0.920	0.34^a^
Negative	530 (92.7)	474 (93.1)	56 (88.9)		
Positive	42 (7.3)	35 (6.9)	7 (11.1)		
**Gonorrhea**				0.138	0.90^a^
Negative	542 (94.8)	483 (94.9)	59 (93.7)		
Positive^b^	30 (5.2)	26 (5.1)	4 (6.3)		
**Chlamydia**				1.274	0.26
Negative	470 (82.2)	415 (81.5)	55 (87.3)		
Positive^c^	102 (17.8)	94 (18.5)	8 (12.7)		
**Anogenital warts**				0.000	1.00^a^
Negative	544 (95.1)	482 (95.1)	60 (95.2)		
Positive	28 (4.9)	25 (4.9)	3 (4.8)		
**Any HIV/STIs**				0.040	0.84
Negative	384 (67.1)	341 (67.0)	43 (68.3)		
Positive	188 (32.9)	168 (33.0)	20 (31.7)		

a Continuity correction Chi-square test.

b Positive for either rectal or urethral gonorrhea or both.

c Positive for either rectal or urethral chlamydia or both.

80.8% (462/572) MSM used at least one GSN app in the past 6 months. MSM used apps in the past 6 months were more likely to be chlamydia positive (19.5 vs. 10.9%, *P* = 0.04). No statistically significant difference was found in the overall prevalence of HIV/STIs (33.3 vs. 30.9%, *P* = 0.63), the prevalence of HIV (8.2 vs. 7.6%, *P* = 0.25), syphilis (7.3 vs. 6.5%, *P* = 0.11), gonorrhea (5.4 vs. 4.5%, *P* = 0.71), and anogenital warts (5.0 vs. 4.5%, *P* = 0.85).

## 4. Discussion

In our study, 89.0% of MSM had ever used GSN apps. Compared with non-app users, app users reported more casual sex partners, unprotective anal sex and were less likely to know their last sex partner's HIV status. In the meantime, app users were more likely to have tested for HIV in the past year and be circumcised. Prevalence of HIV and other STIs did not vary between app users and non-app users.

Our study found that 89.0% MSM had ever used GSN apps, and 63.8% MSM had AI partners found *via* apps in the past 6 months. This was higher than the studies conducted in China found 40.6% MSM used apps to seek sex partners in 2013, and 57.9% in 2014 (12, 23). One explanation may be that the three cities enrolled are metropolitan cities. The attitude toward MSM is more inclusive and open, especially in Guangzhou and Shenzhen. And the recent development of GSN apps such as live streaming services might help expand the coverage of GSN apps among MSM.

Our study did not find an association between app use and HIV or other STIs. This is similar to an online research conducted in China (12). Whereas, a meta-analysis suggested that app use was not associated with HIV and syphilis infections, but app users were more likely to have gonorrhea and chlamydia (13). The possibly reason for the inconsistent findings with our study may be that the meta-analysis only included three studies which accessed self-reported gonorrhea and chlamydia, and all studies were conducted in the United States.

The similar prevalence of HIV between app users and non-app users could be explained as follows. App users could have been more willing to use preexposure prophylaxis (PrEP) and post exposure prophylaxis (PEP) compared with non-app users (28, 29). Although app users were more likely to have CAI, app users were also more likely to tested for HIV. A qualitative study conducted in Hong Kong showed that app users were concerned about acquiring HIV and other STIs after engaging in CAI, and attended clinics to test for HIV and other STIs (30). One possible contributing factor for HIV testing may be that app user commonly had high educational level (13), as in our study 70.7% app users had an educational level of college and above, and had high risk perception of HIV and other STIs after CAI. Our study showed that app users were nearly twice more likely to have been tested for HIV in the past year compared with non-app users. Thus, app users are more likely to know their status of HIV. HIV negative app users may use PrEP since a negative HIV status is required for PrEP initiation, and HIV positive app users may receive a timely treatment, which may lead to decreased transmission. Our study also found app users were five times more likely to be circumcised. A recent systematic review and meta-analysis suggested that circumcision is associated with 23% reduced odds of HIV infection (31). This effect is even more pronounced in MSM who are younger and who engage in insertive anal sex (31). As such, the higher circumcision rate among app users may offset part of the HIV risk. The possible reason for similar prevalence of STIs other than HIV between app users and non-app users may be that app users were more like to tested for STIs, which may facilitate timely treatment. Syphilis, gonorrhea and chlamydia are treatable and timely treatment means less transmission. Meanwhile, the systematic review and meta-analysis found circumcision is associated with 9% reduced odds of any STI other than HIV (31). However, our study was a cross-sectional study, prospective studies are needed to better investigate the impact of app use on HIV/STIs.

Our study found that app users were more likely to have more than one casual sex partners in the past 6 months and have regular sex partners. This is consistent with a previous study that indicated app users were associated with having both stable and casual sex partners (20). One of the primary reasons for MSM to use GSN apps is to find sex partners (32). Given the convenience of GSN apps, it is easy for app users to find casual sex partners. As time goes by, some casual sex partners may develop to regular sex partners. And in this study, 44.1% (124/281) of app users who have regular sex partners were not in romantic relationships with their regular sex partners. They may use GSN apps to seek for extra-relational casual sex partners.

Our study also found that more than half of app users (56.4%) did not know their last sex partner's HIV status. This finding is consistent with a research conducted in Beijing (10). One explanation may be that MSM did not know their own recent HIV status. In this study, 24.4% app users did not test for HIV in the past year. A previous study showed that longer time since last HIV test was associated with decreased HIV status communication with sex partners (33). Another explanation maybe that the last sex partner of app users was more likely to be casual sex partner. A previous study indicated that MSM were more likely to disclose their HIV status to regular sex partners (33). Existing studies indicated that awareness of partner's HIV status increases condom use, which could decrease the risk of HIV acquisition (34, 35). Therefore, more interventions targeting HIV status communication with sex partners among MSM, including HIV self-testing are needed. MSM could know their sex partners HIV status through HIV self-testing, and make safer sex decision.

Our study is one of the few studies about GSN app use and its association with HIV/STIs among MSM that were based on offline recruitment and laboratory testing of HIV/STIs in China. The study preliminarily explores the impact of app use on STIs. Studies comparing the incidence of HIV/STIs between long-term app users and non-app users are needed to provide insight with regard to the true impact of app use on HIV/STIs risk.

The study has several limitations. First, there might be recall bias and social desirability bias regarding self-reported information, especially when stigma surrounding HIV and MSM was existent. In order to minimize these biases, most questions about sexual behaviors and use of apps were limited to the past 6 months, and all questionnaires were anonymous. Second, the number of non-app users only accounted for 11.0% (63/572) of all participants in this study, which may not be well representative of the non-app users. Third, the study was conducted at three metropolitan cities in China. Thus, generalization may be limited to rural areas and other countries. Fourth, the sample was limited to MSM who were literate, able to read/speak Mandarin, and familiar with smart devices or iPads. The study was conducted some 5 years ago. App use behaviors of MSM may have changed over time, especially with the influence of the coronavirus disease 2019 (COVID-19) and technological advances. And this was a cross-sectional study, and thus we were not able to make a causal inference.

## 5. Conclusions

GSN apps have been widely used among MSM in China. Our study suggested that app users were associated with more casual sex partners, unprotective anal sex and unaware their last sex partner's HIV status, but they were more likely to have tested for HIV in the past year and be circumcised. Prevalence of HIV/STIs did not vary between app users and non-app users. Longitudinal studies comparing the incidence of HIV/STIs between long-term app users and non-app users may be necessary to clarify the impact of app use on HIV/STI risk. Researchers and health providers should collaborate with GSN app developers to explore the role of GSN apps in HIV/STIs prevention and control.

## Data availability statement

The original contributions presented in the study are included in the article/supplementary material, further inquiries can be directed to the corresponding author.

## Ethics statement

The studies involving human participants were reviewed and approved by University of New South Wales, Australia, Dermatology Hospital of Southern Medical University, Wuxi Centre for Disease Control and Prevention, China. The patients/participants provided their written informed consent to participate in this study.

## Author contributions

HZ designed the study and supervised data collection. ZG analyzed the data and drafted the manuscript. AF, YZ, YG, YS, YC, and XZ participated in data collection or result interpretation. All authors have approved the submitted versions.
